# Antitumor activity of arsenite in combination with tetrandrine against human breast cancer cell line MDA-MB-231 in vitro and in vivo

**DOI:** 10.1186/s12935-018-0613-0

**Published:** 2018-08-13

**Authors:** Bo Yuan, Mingjiang Yao, Xiao Wang, Ai Sato, Ayane Okazaki, Hana Komuro, Hideki Hayashi, Hiroo Toyoda, Xiaohua Pei, Xiaomei Hu, Toshihiko Hirano, Norio Takagi

**Affiliations:** 1 Department of Applied Biochemistry, School of Pharmacy, Tokyo University of Pharmacy & Life Sciences, 1432-1 Horinouchi, Hachioji, Tokyo 192-0392 Japan; 2 Department of Clinical Molecular Genetics, School of Pharmacy, Tokyo University of Pharmacy & Life Sciences, 1432-1 Horinouchi, Hachioji, Tokyo 192-0392 Japan; 3 XiYuan Hospital, China Academy of Chinese Medical Sciences, Beijing, 100091 People’s Republic of China; 4 The Third Affiliated Hospital of Beijing University of Traditional Chinese Medicine, Beijing, 100029 People’s Republic of China; 5 Department of Clinical Pharmacology, School of Pharmacy, Tokyo University of Pharmacy & Life Sciences, 1432-1 Horinouchi, Hachioji, Tokyo 192-0392 Japan

**Keywords:** Arsenite, Tetrandrine, MDA-MB-231 cells, Cell death, Combination therapy

## Abstract

**Background:**

Triple-negative breast cancer (TNBC) is one of the most difficult subtypes of breast cancer to treat due to its aggressive, metastatic behavior, and a lack of a targeted therapy. Trivalent arsenic derivatives (arsenite, As^III^) with remarkable clinical efficacy in acute promyelocytic leukemia has been demonstrated to exhibit inhibitory effect against breast cancer cells. To provide novel insight into the development of new therapeutic strategies, antitumor activity of As^III^ and tetrandrine (Tetra), a Chinese plant-derived alkaloid, against the TNBC cell line MDA-MB-231 in vitro and in vivo was investigated.

**Methods:**

Cytotoxicity was evaluated using cell viability, lactate dehydrogenase leakage and cell cycle assay. Alterations of genes related to cell proliferation and death were analyzed using western blotting. In vivo antitumor activity of As^III^ alone or in combination with Tetra was studied using MDA-MB-231 xenografts in nude mice.

**Results:**

Synergistic cytotoxic effects of two drugs were observed in the cells. In vivo study also showed that co-administration of As^III^ and Tetra significantly reduced tumor volume and weight, directly supporting its in vitro antitumor activity. No deaths and reduction of body-weight were observed after a long-term co-administration, indicating its good tolerability. S-phase arrest associated with the upregulation of FOXO3a, p27 along with decreased Cyclin D1 expression was observed in the cells treated with the combined regimen. A substantial upregulated p21 expression and downregulated phospho-FOXO3a and Cyclin D1 expression was observed in the tumor tissues of mice co-administered with As^III^ and Tetra. Autophagy induction was observed in the combination treatment in vitro and in vivo. The addition of wortmannin, a potent autophagy inhibitor, significantly rescued MDA-MB-231 cells from their cytotoxicity of As^III^ and Tetra.

**Conclusions:**

S-phase arrest, autophagic and necrotic cell death contribute to the cytocidal effects of the combined regimen of As^III^ and Tetra. Considering our previous study showing synergistic cytotoxic effects of the combined regimen in estrogen receptor-positive breast cancer cell line MCF-7, these results suggest that development of the combination regimen of As^III^ plus Tetra may offer many benefits to patients with different types of breast cancer.

## Background

Breast cancer is the most common cancer among women worldwide and persists as one of the leading causes of cancer-related deaths in women. Three distinct biomarkers including the estrogen receptor (ER), progesterone receptor (PR), and human epidermal growth factor receptor-2 (HER2) are used to determine the appropriate breast cancer therapy [1]. Tamoxifen (Nolvadex^®^) and trastuzumab (Herceptin^®^) have already been successfully applied to patients diagnosed with ER-positive and HER2-positive breast cancer, respectively [2]. Among different types of breast cancer, triple negative breast cancer (TNBC; ER, PR, and HER2-negative breast cancer), accounting for approximately 10–20% of breast cancer cases, is one of the most difficult subtypes of breast cancer to treat due to its aggressive, metastatic behavior, and a lack of a targeted therapy [1]. Therefore, novel therapeutic strategies are urgently needed as most patients with TNBC relapse with distant metastases, and hormonal therapies and HER2-targeted agents are ineffective in this group of tumors.

Administration of trivalent arsenic derivatives (arsenite, As^III^) such as arsenic trioxide (As_2_O_3_) has demonstrated a remarkable efficacy in the treatment of relapsed and refractory acute promyelocytic leukemia (APL) patients. Several research groups including us have conducted detailed pharmacokinetic studies of As^III^ in APL to optimize its treatment [3–6]. We have also investigated the effects of As^III^ using a unique in vitro system comprising primary cultured chorion and amnion cells prepared from human fetal membranes, and demonstrated that aquaporin 9 and multidrug resistance-associated protein 2 are functionally involved in controlling arsenic accumulation in these normal cells, which then contribute to differential sensitivity to As^III^ cytotoxicity between these cells [7]. These findings may provide a new insight into clinical applications of As_2_O_3_, and better therapeutic protocols [8]. At the same time, the successful clinical efficacy of As^III^ in the treatment of APL patients has encouraged further studies on its potential treatment applications for other malignancies, including solid tumors [8, 9]. In fact, As^III^ has been demonstrated to exhibit inhibitory effects against breast cancer cells [10, 11], raising the possibility of repositioning arsenic compounds to treat patients with breast cancer.

Recently, we demonstrated a clear cytotoxic effect of As^III^ against ER-positive human breast cancer cell line MCF-7, and further clarified that tetrandrine (Tetra), a bis-benzylisoquinoline alkaloid isolated from the root of *Stephania tetrandra* S. Moore, significantly enhanced the cytotoxicity of As^III^ in a synergistic manner [12]. QT prolongation is known as a major complication in As^III^ therapy [8], closely related to the intracellular [Ca^2+^] overload induced by As^III^ [13], Tetra, on the other hand, has been demonstrated to serve as a calcium channel antagonist significantly decreasing intracellular [Ca^2+^] within ventricular cells [14]. Therefore, we suggested that the combination regimen of As^III^ and Tetra may be expected not only to achieve improved efficacy of As^III^ in the treatment with ER-positive breast cancer, but also overcome its adverse cardiac effects secondary to Tetra functioning as calcium channel blocker. However, the antitumor activity of As^III^ in combination with Tetra against TNBC cell line MDA-MB-231 in vitro and in vivo has not yet been investigated.

Cell cycle arrest as well as autophagic cell death has been considered as the major underlying mechanisms of action of most anticancer drugs [11, 15–19]. The cell cycle is known to be precisely regulated by a number of vital molecules known as cyclin-dependent kinases (CDKs) and CDK inhibitors such as p21 Waf1/Cip1 (p21) and p27 Kip1 (p27) [11, 20, 21]. Forkhead box transcription factor (FOXO3a), which is considered to be involved in the development of breast cancer and may also serve as its prognostic marker [22], has been linked to the regulation of genes involving multiple cellular processes such as cell cycle, invasion, and cell death [21–24]. FOXO3a is also known to be targeted for degradation by phosphorylation [25, 26]. Phosphorylation of FOXO3a will results in its nuclear export and thereby consequent degradation, and consequently interfered with its function as tumor suppressor [25, 26]. Upregulation of p21 and p27 associated with the increased FOXO3a expression has been demonstrated to be responsible for G_0_/G_1_ cell cycle arrest of MCF-7 [12], while their alterations has also been implicated in S-phase arrest in various types of cancer cells including another TNBC cell line Hs578T [27–30]. These differential cell cycle responses may be attributed to different cell types and/or genetic and phenotypic diversity of cancer cells. However, whether and how these molecules contribute to the potential cytotoxic effects induced by the combination of As^III^ and Tetra against MDA-MB-231 in vitro and in vivo remain to be seen.

In this study, antitumor activity of As^III^ in combination with Tetra against the TNBC cell line MDA-MB-231 in vitro and in vivo was investigated by focusing on cell cycle arrest and autophagic cell death. Key regulatory molecules associated with the cell cycle and death were investigated to further elucidate cytotoxic mechanisms.

## Materials and methods

### Materials

Sodium arsenite (NaAsO_2_, As^III^) and tetrandrine (Tetra) were purchased from Tri Chemical Laboratories (Yamanashi, Japan) and National Institutes for Food and Drug Control (Beijing, China), respectively. Fetal bovine serum (FBS) was purchased from Nichirei Biosciences (Tokyo, Japan). Dulbecco’s modified Eagle’s medium (DMEM), phenazine methosulfate (PMS) and dimethyl sulfoxide (DMSO) were obtained from Wako Pure Chemical Industries (Osaka, Japan). Wortmannin, a potent autophagy inhibitor, propidium iodide (PI), ribonuclease A (RNaseA) and 2,3-bis(2-methoxy-4-nitro-5-sulfophenyl)-5-[(phenylamino)carbonyl]-2*H*-tetrazolium hydroxide (XTT) were purchased from Sigma-Aldrich (St. Louis, MO, USA). Tetrandrine hydrochloride injection and arsenious acid and sodium chloride injection were obtained from Jiangxi Yintao Pharmaceutical Co., Ltd. (Jiangxi, China) and Heilongjiang Harbin Pharmaceutical Co., Ltd. (Heilongjiang, China), respectively for the in vivo study. Can Get Signal^®^ Immunoreaction Enhancer Solution were purchased from Toyobo Co., Ltd. (Osaka, Japan).

### Cell culture and treatment

MDA-MB-231 cells were obtained from the American Type Culture Collection (ATCC, Manassas, VA, USA). Cells were cultured in DMEM medium supplemented with 10% heat-inactivated FBS and 100 U/ml of penicillin and 100 µg/ml of streptomycin in a humidified 5% CO_2_ atmosphere at 37 °C. The cells were seeded in 96-well plates (Iwaki, Tokyo, Japan) at a density of 1 × 10^4^ cells/well in 0.1 ml medium and cultivated for 24 h. Cultures in sixplicate were treated with various concentrations of As^III^ and Tetra, alone or in combination.

### Cell viability assay

The cytotoxicity of As^III^ and Tetra, alone or in combination, to MDA-MB-231 cells was measured by XTT dye-reduction assay according to the method previously described with slight modifications [7, 12]. Briefly, after treatment with As^III^ and Tetra, alone or in combination, for 48 h, XTT and PMS were added into each well at final concentrations of 0.2 mg/ml and 1 mM, respectively. After incubation at 37 °C for 4 h, the plates were mixed and the absorbance at 450 nm was measured with a microplate reader (EMax Plus^®^, Molecular Devices, CA, USA). The relative cell viability was expressed as the ratio of the absorbance of each treatment group against those of the corresponding untreated control group. Data are shown as mean ± standard deviation (SD) from more than three independent experiments. The IC_50_ values of As^III^ and Tetra were calculated using GraphPad Prism^®^ 6 software. In order to evaluate whether the two drugs generated synergistic, antagonistic, or additive effects, a combination index (CI) was determined as reported previously, using the computer software ComboSyn (Combosyn Inc. NJ, USA) for drug combinations and for general dose–effect analysis, which was developed by Chou [31, 32]. The effect of the combination treatment was defined as a synergistic effect if CI < 1, an additive effect if CI = 1 or an antagonistic effect if CI > 1 [12, 15].

### Antitumor activity of As^III^ alone or in combination with Tetra in MDA-MB-231 mouse xenografts

In vivo antitumor activity of As^III^ alone or in combination with Tetra was studied using human breast cancer nude mouse xenograft model. 5-week-old female immunodeficient BALB/c nude mice were obtained from Japan SLC, Inc. (Shizuoka, Japan) and housed at 23 ± 1 °C in a room with a constant humidity of 55 ± 5% and a regular 12-h light/12-h dark cycle for several days. MDA-MB-231 cells [1 × 10^7^, suspended in 0.1 ml phosphate-buffered saline (PBS)] were then injected subcutaneously into the right flank of each mouse. Tumors (visualized as a small nodule with approximate size of 20–30 mm^3^ at the sites of injection) appeared approximately 5–7 days later after injection, and the mice were randomly divided into four groups (n = 5) according to body weight and tumor size using SPSS 21.0 software, and given the following treatments: vehicle-control (treated with PBS); As^III^ alone (2 mg/kg/day); Tetra alone (20 mg/kg/day); As^III^ (2 mg/kg/day) + Tetra (20 mg/kg/day). The mice were administered i.p. as described above once a day for 10 weeks, respectively. The tumor size was measured every day in two perpendicular dimensions with vernier calipers, and the tumor volume (TV) (mm^3^) was calculated by the formula reported previously [33]: TV = length (mm) × width^2^ (mm^2^) × 0.5. The body weights were also measured every day and were used as an indicator of systemic toxicity of the treatment. Throughout the experiment, the mice had free access to food and water according to the National Institute of Health Guide for the Care and Use of Laboratory Animals and the Guidance for Experimental Animal Care issued by the Prime Minister’s Office of Japan. At the end of the treatment, all animals were sacrificed, and the tumors were removed, photographed and weighed. Tumors were fixed in 4% paraformaldehyde (PFA) in 0.1 M phosphate buffer or frozen in liquid nitrogen for further analysis. The study was approved by the Committee of Animal Care and Welfare of Tokyo University of Pharmacy and Life Sciences.

### Cell cycle analysis

After treatment with the indicated concentrations of As^III^ and Tetra, alone or in combination, for 48 h, cell cycle analysis was performed using a FACSCanto flow cytometer (Becton–Dickinson, CA, USA) according to a method reported previously [12, 34]. Briefly, cells were washed twice with PBS, fixed with 1% paraformaldehyde/PBS for 30 min, washed twice again with PBS, permeabilized in 70% (v/v) cold ethanol and kept at − 20 °C for at least 4 h. Cell pellets were then washed twice with PBS after centrifugation and incubated with 0.25% Triton-X 100 for 5 min on ice. After centrifugation and washing with PBS, cells were resuspended in 500 µl of PI/RNase A/PBS (5 µg/ml of PI and 0.1% RNase A in PBS) and incubated for 30 min in the dark at room temperature. A total of 10,000 events were acquired and Diva software and ModFit LT™ Ver.3.0 (Verity Software House, ME, USA) were used to calculate the number of cells at each G_0_/G_1_ and S phase fraction.

### Lactate dehydrogenase (LDH) assay

Since the combined treatment of the relatively low concentration of 10 µM As^III^ and 4 µg/ml Tetra achieved appropriate level of the cytocidal effect in MDA-MB-231 cells, the above-mentioned concentrations were used to conduct LDH assay. After treatment with 10 µM As^III^ and 4 µg/ml Tetra, alone or in combination, for 48 h, LDH leakage from cells was measured using the LDH-Cytotoxic Test Wako kit (Wako Pure Chemical Industries, Osaka, Japan) according to the method previously described with slight modifications [7, 12]. Briefly, culture supernatants were collected by centrifugation at 2500 rpm for 5 min at 4 °C. Non-treated cells were lysed in culture medium containing 0.2% Tween 20, and mixed aggressively using a vortex mixer, followed by the centrifugation at 12,000×*g* for 10 min and the cell lysate was used as the positive control. Culture medium served as the negative control. Culture supernatants were collected then diluted 16-fold with PBS and 50 μl of the diluted solution was transferred into wells of a 96-well plate. LDH activities were determined by adding 50 μl of ‘substrate solution’ from the kit, followed by incubation at room temperature for 30 min. The reaction was stopped by the addition of 100 μl of ‘stopping solution’ and the absorbance at 560 nm was measured with a microplate reader (Safire, Tecan, Switzerland). Cell damage was calculated as a percentage of LDH leakage from damaged cells using the following formula:

$${\text{LDH}}\,{\text{leakage}}\left( \% \right) = \left( {{\text{Sup}} - {\text{NC}}} \right)/\left( {{\text{P}} - {\text{NCT}}} \right) \times 100$$

where Sup, NC, P and NCT refer to the absorption of the culture supernatant, negative control, positive control and culture medium containing 0.2% Tween 20, respectively. In order to evaluate the correlation between necrosis and autophagy induction, cells were treated with 0.25 μM wortmannin for 30 min prior to the treatment with 10 μM As and 4 μg/ml Tetra, alone or in combination, in the presence or absence of 0.25 μM wortmannin for an additional 48 h, followed by LDH leakage assay as described above.

### Western blot analysis

For preparation protein samples, cell pellets (approximately 1–2 × 10^6^ cells per 110 μl Laemmli buffer) and tumor tissues (at a ratio of approximately 1 g of tissue per 10 ml Laemmli buffer) obtained from MDA-MB-231 mouse xenografts were suspended in Laemmli buffer containing 100 mM DTT, 2 μg/ml leupeptin, 2 μg/ml aprotinin, 1 μg/ml pepstatin, 1 mM PMSF. The suspensions of cells and tumor tissues were sonicated using a sonicator (Qsonica, LLC, CT, USA) with 10 short burst of 2 s followed by intervals of 2 s for cooling. The suspensions were kept at all times in an ice bath. Sonicated cells and tumor tissues were heated in 95 °C for 5 min, and then centrifuged at 13,000*g* for 15 min at 4 °C. Protein concentrations of the supernatant were determined according to Bradford’s method using the protein assay dye reagent (Bio-Rad, CA, USA) according to the manufacturer’s instructions, and using BSA as the standard. Western blot analysis was carried out according to the methods previously described [35]. Briefly, after separation of proteins on a sodium dodecyl sulfate (SDS) polyacrylamide gel electrophoresis, followed by transferring to a polyvinylidene difluoride (PVDF) membrane (Millipore Corp, MA, USA), protein bands were detected using the following primary antibodies and dilution ratios: mouse anti-human β-actin (1:5000 dilution; cat. no. A-5441; Sigma-Aldrich, MO, USA); rabbit anti-human phospho-FOXO3a (Ser253) (1:1000 dilution; cat. no. 9466) and FOXO3a (1:1000 dilution; cat. no. 2497), rabbit anti-human p27 (1:1000 dilution; cat. no. 2552), mouse anti-human p21 (1:1000 dilution; cat. no. 2946), rabbit anti-human Cyclin D1 (1:1000 dilution; cat. no. 2978), rabbit anti-human phospho-AMPKα1 (Ser485) (1:1000 dilution; cat. no. 2537) and AMPKα (1:1000 dilution; cat. no. 2532), rabbit anti-human phospho-mTOR (Ser2448) (1:1000 dilution; cat. no. 5536) and mTOR (1:1000 dilution; cat. no. 2983), rabbit anti-human Beclin-1 (1:1000 dilution; cat. no. 3495), rabbit anti-human LC3 (1:1000 dilution; cat. no. 12741) (Cell Signaling Technology, MA, USA). Blotted protein bands were detected with respective horseradish peroxidase-conjugated secondary antibody and an enhanced chemiluminescence (ECL) Western blot analysis system (Amersham Pharmacia Biotech, Buckinghamshire, UK).

### Statistical analysis

Experiments were independently repeated three times, and reported as the mean ± SD of the three assays. Statistical analysis was conducted using one-way ANOVA followed by Dunnett’s post-test. A probability level of p < 0.05 was considered statistically significant.

## Results

### In vitro and in vivo growth inhibition of human breast cancer cell line MDA-MB-231 by As^III^ and Tetra, alone or in combination

**Fig. 1:**
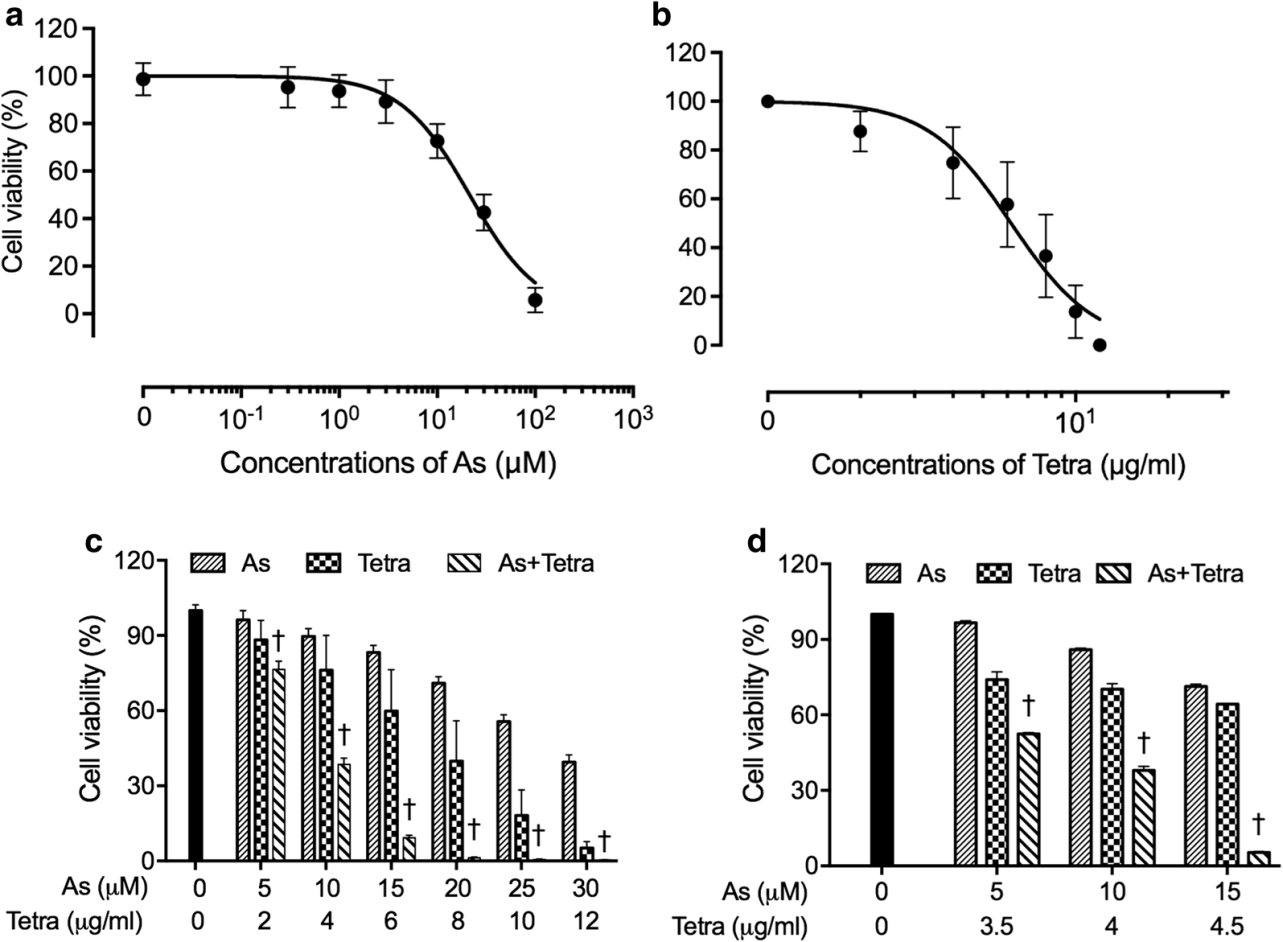
Cytotoxic effect of As^III^ and Tetra, alone or in combination, against human breast cancer cell line MDA-MB-231. Cell viability was determined by XTT assay after the treatment with various concentrations of As^III^ alone (0.3, 1, 3, 10, 30 and 100 µM) (**a**), Tetra alone (2, 4, 6, 8, 10 and 12 µg/ml) (**b**), their combination in constant ratio (5 µM As^III^ + 2 µg/ml Tetra, 10 µM As^III^ + 4 µg/ml Tetra, 15 µM As^III^ + 6 µg/ml Tetra, 20 µM As^III^ + 8 µg/ml Tetra, 25 µM As^III^ + 10 µg/ml Tetra, and 30 µM As^III^ + 12 µg/ml Tetra) (**c**), or combination treatment with relatively low concentrations (5 µM As^III^ + 3.5 µg/ml Tetra, 10 µM As^III^ + 4 µg/ml Tetra, 15 µM As^III^ + 4.5 µg/ml Tetra) (**d**) for 48 h. Relative cell viability was calculated as the ratio of the absorbance at 450 nm of each treatment group against those of the corresponding untreated control group. Data are shown as the means and SD from more than three independent experiments. ^†^*p *< 0.05 vs. each alone. As, As^III^; Tetra, tetrandrine; As + Tetra, As^III^ + tetrandrine

A significant decrease in cell viability was observed in a dose-dependent manner in MDA-MB-231 cells after treatment with various concentrations of As^III^ or Tetra alone for 48 h (Fig. 1a, b), and the IC_50_ values were 19.2 ± 2.6 µM and 6.2 ± 0.2 µg/ml for As^III^ and Tetra treatment, respectively. Next, two-drug combination in constant ratio were designed according to the median-effect method of Chou [31, 32] to evaluate if the two drugs generated synergistic, antagonistic, or additive cytotoxic effects against the cells. As shown in Fig. 1c, the combined treatment was significantly more cytotoxic than either drug alone (p < 0.05). The values of combination index (CI) were < 1, indicating the two drugs worked in a synergistic manner (Table 1). Based on the above-described IC_50_ values of each drug, relatively low different concentrations of each drug (5, 10 and 15 µM for As^III^, and 3.5, 4, and 4.5 µg/ml for Tetra) were designed to generate appropriate efficacy of drug combination in order to clarify the mechanisms underlying the cytocidal effect of the combination of As^III^ and Tetra in detail. Again, synergistic cytotoxic effects of the two drugs were confirmed (Fig. 1d and Table 2).

**Table 1:** CI values of As^III^ at concentrations in combination with Tetra in MDA-MB-231 cells

As (μM)	Tetra (μg/ml)	Fa	CI value
5	2	0.2352	0.89233
10	4	0.6149	0.87988
15	6	0.9077	0.60981
20	8	0.9878	0.33548
25	10	0.9947	0.29544
30	12	0.9968	0.28727

**Table 2:** CI values of As^III^ at concentrations in combination with Tetra in MDA-MB-231 cells

As (μM)	Tetra (μg/ml)	Fa	CI value
5	3.5	0.47567	0.83571
10	4	0.62086	0.87539
15	4.5	0.94673	0.36602

**Fig. 2:**
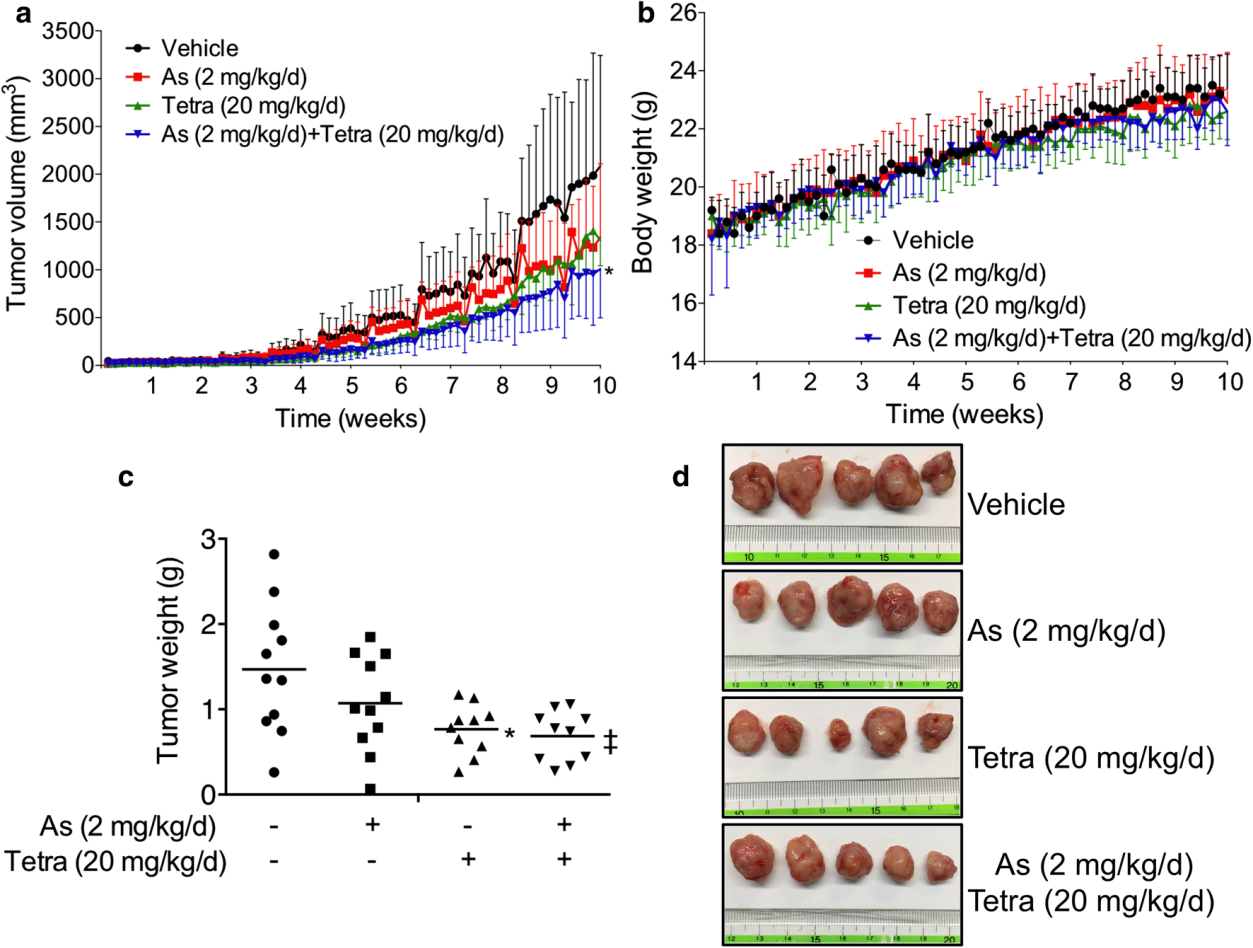
Antitumor activity of As^III^ alone and in combination with Tetra in MDA-MB-231 mouse xenografts. The experiment was carried out using human breast cancer nude mice implanted subcutaneously with 1 × 10^7^ MDA-MB-231 cells. The administration of As^III^ and Tetra, alone or in combination, was performed as described under “Materials and methods”. Tumor volumes (**a**) and body weights (**b**) were measured at indicated time points, respectively. Tumor weights at time of sacrifice (**c**), and photographs of isolated tumors derived from vehicle-control and treated mice (**d**) were presented. *p < 0.05, ^‡^*p *< 0.01 vs. vehicle-control (treated with PBS). As, As^III^; Tetra, tetrandrine; As + Tetra, As^III^ + tetrandrine

The antitumor activity of As^III^ alone or in combination with Tetra was further evaluated in vivo with MDA-MB-231 human breast cancer xenograft model. As shown in Fig. 2a, a reduction in tumor volume was observed in the mice treated by a single administration of As^III^ or Tetra alone, and further strengthened by their combination. Compared to vehicle-control group, tumor volume was reduced by 35.8, 37.2, and 51.6% for As^III^ (2 mg/kg/day) alone, Tetra (20 mg/kg/day) alone, and their combination, respectively, at the end of experiments (week 10 point). Consistently, treatment with As^III^ and Tetra, alone or in combination, also led to reduction of tumor weight. Tumor weights were 1.47 ± 0.76, 1.07 ± 0.56, 0.77 ± 0.29 and 0.69 ± 0.29 g in the vehicle-control group, As^III^ alone, Tetra alone and co-administration groups at week 10 after implantation, respectively (Fig. 2c, d). Of note, no alteration in the body weight was observed in mice between vehicle-control group and treatment group regardless of the long-term administration of either drug alone or their combination (Fig. 2b), indicating the combined treatment was well tolerated by all mice.

### Effects of As^III^ and Tetra, alone or in combination, on the cell cycle profiling and the expression level of cell cycle related-proteins in MDA-MB-231 cells

**Fig. 3:**
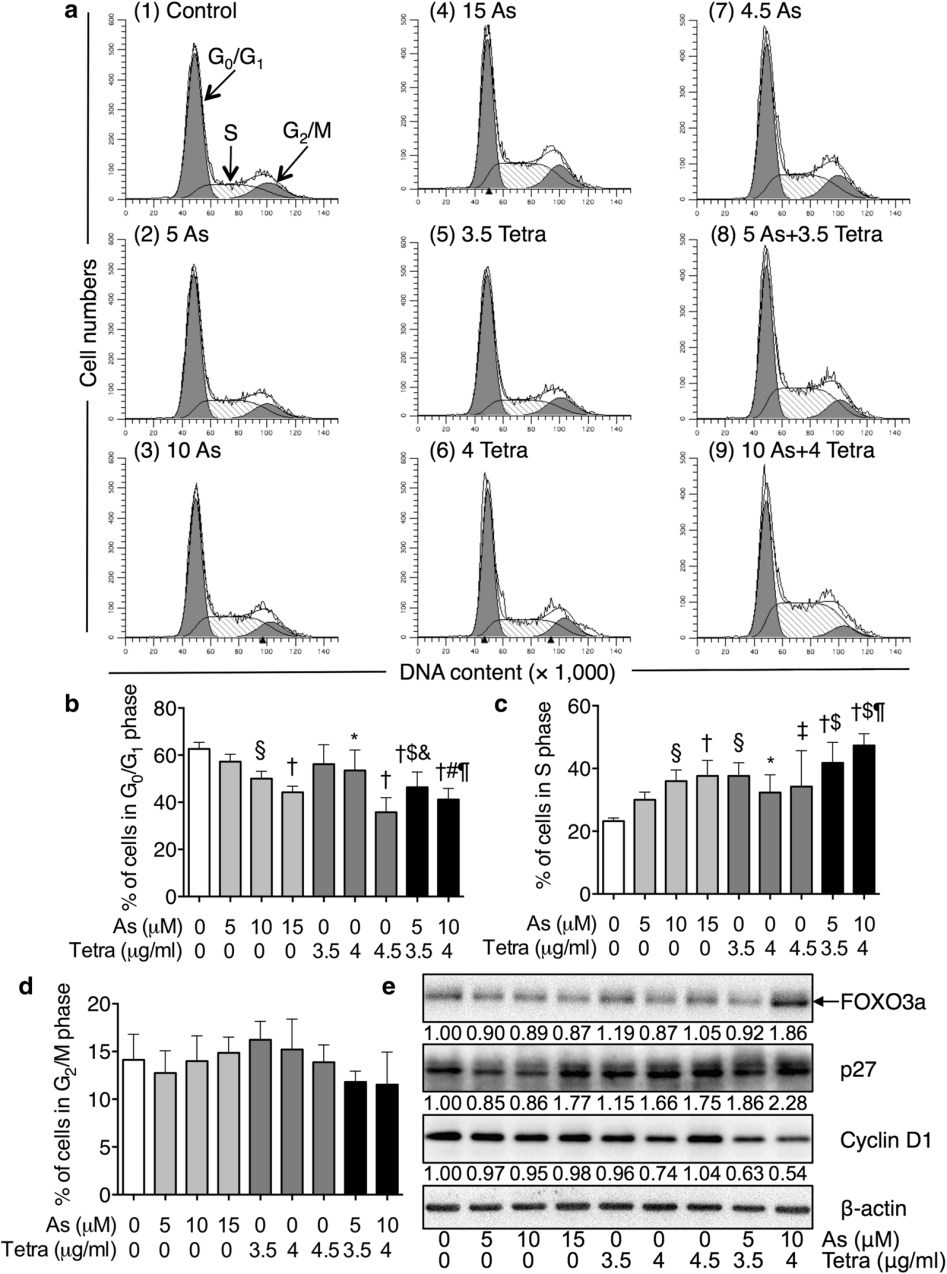
Effects of As^III^ and Tetra, alone or in combination, on the cell cycle profiling and the expression level of cell cycle related-proteins in MDA-MB-231 cells. **a**–**d** After treatment with various concentrations of As^III^ (5, 10 and 15 µM), and Tetra (3.5, 4 and 4.5 µg/ml), alone or in combination (5 µM As^III^ + 3.5 µg/ml Tetra, 10 µM As^III^ + 4 µg/ml Tetra), for 48 h, cell cycle profiling was performed by FACSCanto flow cytometer as described under “Materials and methods”. Analyzed data and profiles for each G_0_/G_1_ and G_2_/M phase using Diva software and ModFit LT™ ver. 3.0. are shown in the gray area. Cells at S phase are shown as shaded area. A representative FACS histogram from three separate experiments is shown. Significant difference between control and treatment with As^III^ and Tetra, alone or in combination, are shown (*p < 0.05, ^‡^*p *< 0.01, ^§^*p *< 0.001, ^†^*p *< 0.0001 vs. control, ^#^p < 0.05, ^$^*p *< 0.01 vs. As^III^ alone; ^&^p < 0.05, ^¶^p < 0.01 vs. Tetra alone). **e** Representative image of the expression profile of each protein is shown from three independent experiments. The densitometry of protein bands was analyzed using a program, NIH ImageJ 1.52a. The values under each image represent the ratios between each key molecule and β-actin protein expression levels, which were further compared with those of control group (untreated cells). As, As^III^; Tetra, tetrandrine. Since enough cells cannot be collected in the group treated with 15 µM As^III^ in combination with 4.5 µg/ml Tetra due to its strong cytotoxicity, cell cycle and western blot analysis were not conducted

To explore whether cell cycle arrest is involved in the cytotoxic effect of As^III^ and Tetra, cell cycle analysis was performed using flow cytometry. As shown in Fig. 3a, c, after treatment with various concentrations of As^III^ and Tetra, alone or in combination, for 48 h, a significant increase in the number of cells in S phase was induced by As^III^ alone and Tetra alone. Furthermore, a significant increase in the number of cells in S phase was induced by the combined treatment in comparison to that induced by either drug alone. Concomitantly, a significant decrease in the number of cells in G_0_/G_1_ phase was also observed (Fig. 3a, b). Almost no alteration was observed in the number of cells in G_2_/M phase was observed (Fig. 3a, d). As shown in Fig. 3e, in comparison to control group, the expression of FOXO3a was clearly upregulated by the combined treatment of 10 μM As^III^ and 4 μg/ml Tetra, although almost no alteration was observed in the cells treated with As^III^ and Tetra, each alone. The expression level of p27 was increased by the highest concentrations of As^III^ (15 μM As^III^) and Tetra alone compared to the control, and a modest but clear enhancement in its expression was observed in the combined treatment group, especially in the combined regimen of 10 μM As^III^ and 4 μg/ml Tetra. Although only a modest decrease in the expression level of Cyclin D1 was observed when treated with As^III^ and Tetra, each alone, a substantial decrease in its expression was confirmed in the combined treatment group.

### Enhanced LDH release in MDA-MB-231 cells treated with As^III^ combined with Tetra

**Fig. 4:**
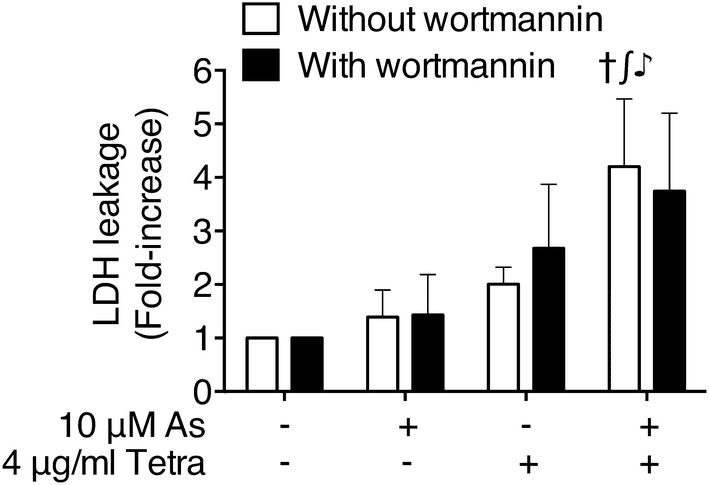
Enhanced LDH release in MDA-MB-231 cells treated with As^III^ combined with Tetra. After treatment with 10 μM As^III^ and 4 μg/ml Tetra, alone or in combination, in the presence or absence of 0.25 μM wortmannin, for 48 h, LDH leakage was measured using the LDH-Cytotoxic test kit as described under “Materials and methods”. Significant difference between control and treatment with As^III^ and Tetra, alone or in combination, are shown (^†^*p *< 0.0001 vs. control; ^∫^*p *< 0.0001 vs. As^III^ alone; ^♪^*p *< 0.001 vs. Tetra). As, As^III^; Tetra, tetrandrine

The release of LDH provides an accurate measure of the cell membrane integrity and cell viability [7, 12]. After treatment with 10 μM As^III^ and 4 μg/ml Tetra, alone or in combination, for 48 h, LDH leakage analysis was thus performed to examine whether the treatments affected cell membrane integrity. As shown in Fig. 4, a non-significant increase in the LDH leakage was observed in MDA-MB-231 cells treated with either As^III^ or Tetra alone compared to the control. A significant enhancement in the LDH leakage was further observed in the combined treatment group, indicating the involvement of necrosis in the cytotoxic effect of As^III^ and Tetra.

### Effect of wortmannin on the cytotoxicity of the combined treatment of As^III^ and Tetra in MDA-MB-231 cells

**Fig. 5:**
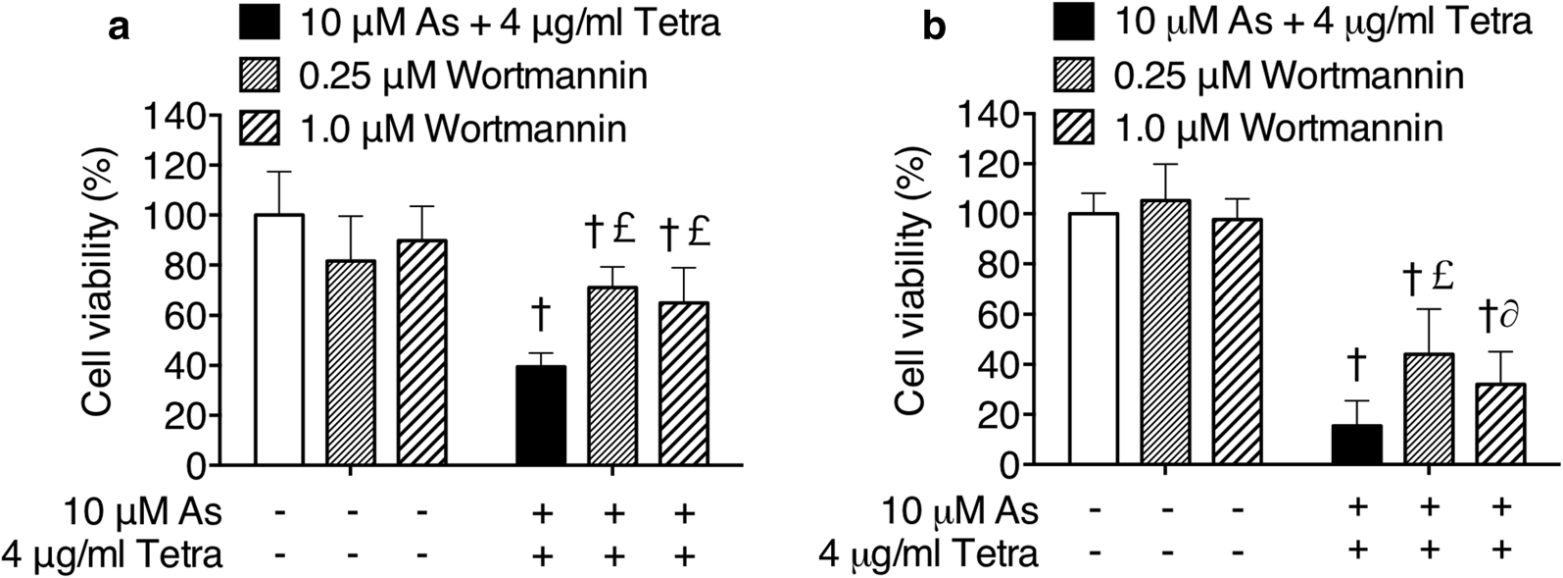
Effect of wortmannin on the cytotoxicity of the combined treatment of As^III^ and Tetra. After the treatment with 10 μM As^III^ combined with 4 μg/ml Tetra in the presence or absence of 0.25 μM and 1.0 μM wortmannin, respectively, for 24 h (**a**) and 48 h (**b**), cell viability was determined by XTT assay as described under “Materials and methods”. Relative cell viability was calculated as the ratio of the absorbance at 450 nm of each treatment group against those of the corresponding untreated control group. ^†^*p *< 0.0001 vs. control; ^∂^*p *< 0.05, ^£^*p *< 0.0001 vs. As^III^ + Tetra. As, As^III^; Tetra, tetrandrine

Since induction of autophagy by various anticancer drugs has been suggested to be a potential therapeutic strategy for cancer including breast cancer [17, 36, 37], a potent autophagy inhibitor, wortmannin was used to investigate whether the induction of autophagy contributed to the combined treatment-induced cytotoxicity. As shown in Fig. 5, a significant decrease in cell viability was observed in MDA-MB-231 cells after treatment with 10 μM As^III^ combined with 4 μg/ml Tetra for 24 h (Fig. 5a) and 48 h (Fig. 5b), respectively. The addition of either 0.25 or 1.0 μM wortmannin, however, significantly rescued cell viability from 39.5 ± 5.4 to 71.0 ± 8.3% and 65.0 ± 13.8%, respectively, for 24 h, and from 15.5 ± 10.0 to 44.0 ± 18.1% and 32.0 ± 13.1%, respectively, for 48 h. It was also confirmed that cell viability was almost not altered by wortmannin alone in the cells. These results indicated the involvement of autophagic cell death in the cytotoxicity of the combination of As^III^ and Tetra. In order to explore a correlation of autophagy with necrosis, the influence of wortmannin on the alterations of LDH leakage in the cells treated with a combination of As^III^ and Tetra was evaluated. No any influence of wortmannin on LDH leakage (Fig. 4) was observed, indicating that autophagy and necrosis independently contribute to the cytotoxicity of the combination of As^III^ and Tetra.

### Activation of autophagic pathway in MDA-MB-231 cells treated with As^III^ and Tetra, alone or in combination

**Fig. 6:**
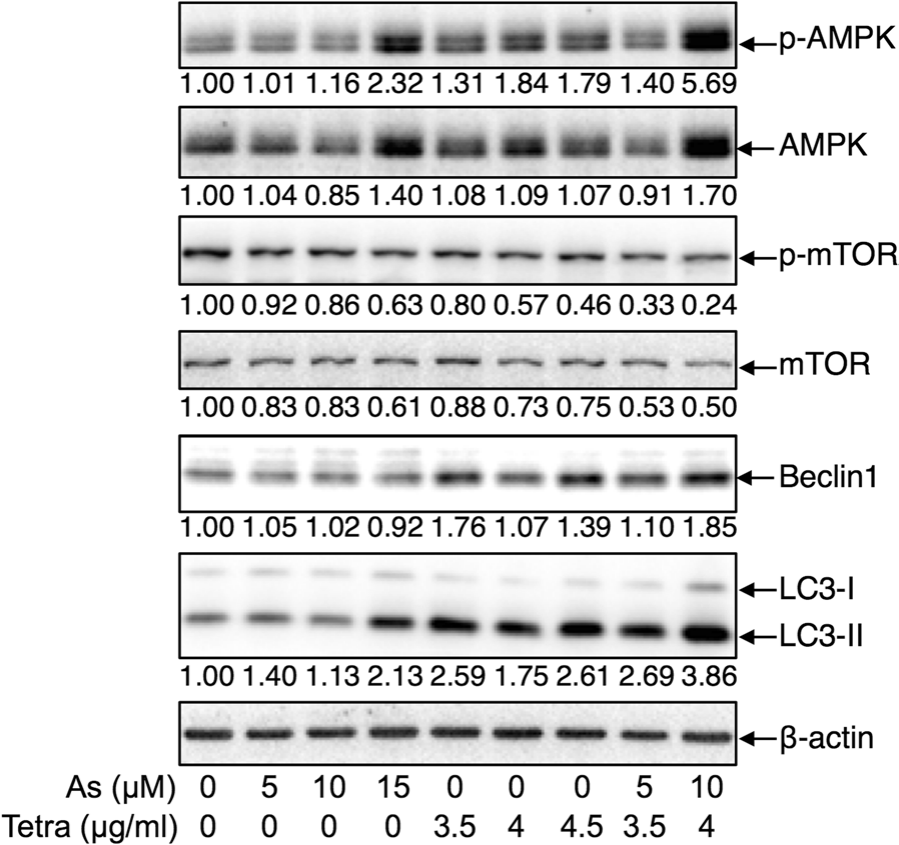
Expression profile of autophagy-related proteins in MDA-MB-231 cells treated with As^III^ and Tetra, alone or in combination. After treatment with various concentrations of As^III^ and Tetra, alone or in combination, for 48 h, the expression profile of autophagy-related proteins was analyzed using western blot as described in “Materials and methods”. Representative image of the expression profile of each protein is shown from three independent experiments. The densitometry of protein bands was analyzed using a program, NIH ImageJ 1.52a. The values under each image represent the ratios between each key molecule and β-actin protein expression levels, which were further compared with those of control group (untreated cells). As, As^III^; Tetra, tetrandrine. Since enough cells cannot be collected in the group treated with 15 µM As^III^ in combination with 4.5 µg/ml Tetra due to its strong cytotoxicity, western blot analysis were not conducted

As shown in Fig. 6, the expression level of LC3, an autophagic marker, was dramatically upregulated by Tetra. Although a relatively low concentration of As^III^ (5 and 10 μM) did not affect the expression level of LC3, a clear increase in its expression level was observed when treated with the highest concentration of 15 μM As^III^. Consistent with the reduction of cell viability induced by 10 μM As^III^ in combination with 4 μg/ml Tetra, and its restoration by the addition of wortmannin (Fig. 6), an obvious upregulation of LC3 expression was observed in the combined treatment in comparison to the treatment with either drug alone. In order to evaluate the mechanisms responsible for the signaling pathway activating autophagy, the expression of a number of autophagy-related proteins was evaluated. Similar to the alterations of the expression levels of LC3, the expression levels of phosphorylated of AMP-activated protein kinase (AMPK) (phospho-AMPK), a key energy sensor and an upstream promoter of autophagy induction [23], was modestly upregulated by Tetra and the highest concentrations of 15 μM As^III^. Again, compared to the treatment with either 10 μM As^III^ or 4 μg/ml Tetra alone, a large enhancement in the expression of phospho-AMPK was observed in their combination treatment. Although treatment with either As^III^ or Tetra alone, except for 15 μM As^III^, almost did not affect the expression levels of total-AMPK, its enhanced expression level was further confirmed in the cells treated with the combination of 10 μM As^III^ and 4 μg/ml Tetra. The alteration of the expression levels of phosphorylated mammalian target of rapamycin (phospho-mTOR) and total-mTOR demonstrated an almost opposite behavior, showing a downregulation of their expression in the treated cells as compared to controls, especially in the combined treatment of 10 μM As^III^ and 4 μg/ml Tetra. These results indicating that AMPK-mediated mTOR deactivation is involved during the autophagy induction. The expression levels of Beclin-1, an autophagic mediator which is deleted in 50% of breast tumors [19], were modestly but clearly upregulated in the cells treated with Tetra alone, but not by As^III^ alone. Notably, the increase in the expression of Beclin-1 by 4 μg/ml Tetra was slightly strengthened by the addition of 10 μM As^III^.

### Expression profile of autophagy and cell cycle arrest-related proteins in MDA-MB-231 mouse xenografts treated with As^III^ and Tetra, alone or in combination

**Fig. 7:**
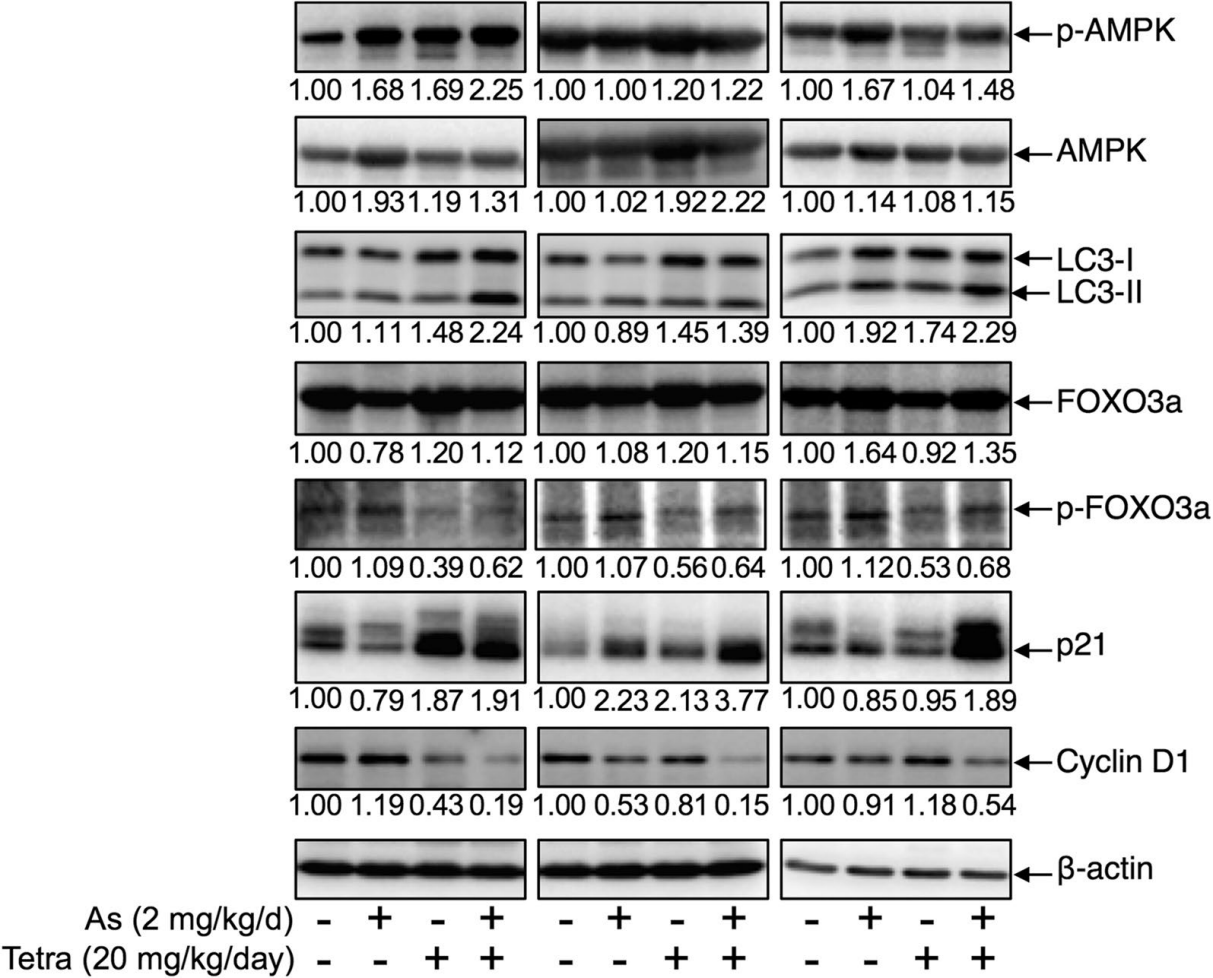
Expression profile of autophagy and cell cycle arrest-related proteins in MDA-MB-231 mouse xenografts treated with As^III^ and Tetra, alone or in combination. The experiment was carried out using human breast cancer nude mice implanted subcutaneously with 1 × 10^7^ MDA-MB-231 cells. The administration of As^III^ and Tetra, alone or in combination, and the preparation of tumor tissues and their protein samples were carried out as described under “Materials and methods”. Results are representatives of three independent experiments. The densitometry of protein bands was analyzed using a program, NIH ImageJ 1.52a. The values under each image represent the ratios between each key molecule and β-actin protein expression levels, which were further compared with those of vehicle-control group

As shown in Fig. 7, the expression level of LC3 was modestly and clearly upregulated by either As^III^ or Tetra alone. Consistent with the in vitro study, the upregulation was further strengthened by their co-administration. The expression levels of phospho-AMPK and total-AMPK were slightly increased by either As^III^ or Tetra alone, and a further increase in its expression level was observed in the co-administered group. A slight increase in the expression levels of FOXO3a was observed in the both single-drug treatment and co-administration group. At the same time, the expression of phospho-FOXO3a (p-FOXO3a) was prominently downregulated by Tetra alone, although only a modest increase in its expression level was observed in As^III^-treatment group. Intriguingly, the modest increase in the expression of p-FOXO3a induced by As^III^ was successfully corrected by the addition of Tetra. Of note, in line with the in vitro study showing that S-phase arrest was induced by As^III^ or Tetra alone, and further strengthened by their combination in MDA-MB-231 cells (Fig. 3), the expression levels of p21 were prominently induced by the co-administration of As^III^ and Tetra. Concomitantly, a substantial decrease in the expression levels of Cyclin D1 was confirmed in the co-administered group, similar to the phenomena observed in MDA-MB-231 cells treated with As^III^ combined with Tetra (Fig. 3e).

## Discussion

Results from this study clearly demonstrated that Tetra significantly enhanced the cytotoxicity of As^III^ against TNBC cell line MDA-MB-231 in a synergistic manner as evidenced by the XTT assay (Fig. 1 and Tables 1, 2). In agreement with our observations, Tetra has been shown to augment the cytocidal effects of chemotherapeutic agents, including As^III^, in different types of solid tumor cells [15, 38]. In addition, our in vivo study demonstrated that compared to vehicle-control group, tumor growth delay was observed after a single administration of As^III^ or Tetra (Fig. 2). Co-administration of As^III^ and Tetra further significantly reduced tumor volume and tumor weight by more than 50% (Fig. 2), directly supporting the in vitro antitumor activity of As^III^ in combination with Tetra. The discrepancy in the antitumor activity of As^III^ and Tetra between in vitro and in vivo might be attributed to the distribution of the two drugs in different organs including tumor tissue of the mice bearing MDA-MB-231 cells xenografts, since the metabolism and distribution of the two drugs in vivo are considered to be more complex than in vitro. Further investigation into a correlation between the distribution of the two drugs and their anti-tumor activity in the MDA-MB-231 breast cancer xenograft model is ongoing in our laboratory.

More importantly, remarkable tolerance to the long-term co-administration of As^III^ with Tetra was confirmed in all mice as evidenced by the fact that the treatment did not cause death among the mice and decrease their body weight. In line with our findings, Tetra has been demonstrated to potentiate the antitumor activity of doxorubicin without a significant increase in toxicity in mice bearing the multidrug resistance (MDR) MCF-7/adr cell xenografts [39]. Tetra combined with daunorubicin, etoposide and cytarabine has also been used to treat the patients with refractory and relapsed acute myeloid leukemia in a multicenter clinical trial. Of 36 patients, 16 (44%) achieved complete remission, 9 (25%) achieved partial remission, and no increase of side effects was observed [40]. In conventional therapy for patients with breast cancer, estrogen receptor is the necessary molecule requisite for the treatment with the antiestrogen tamoxifen [24]. Recently, we have demonstrated similar synergistic cytotoxic effects of As^III^ and Tetra against ER-positive human breast cancer cell line MCF-7 [12]. On the other hand, TNBC cell line MDA-MB-231 is an estrogen independent cell line that does not depend on estrogen for growth and survival [41]. Taking these previous results and our observations into account, we suggest that Tetra can be a useful combination anticancer agent to enhance the therapeutic effect of As^III^ for patients with different types of breast cancers regardless of their estrogen dependency.

We next demonstrated that a clear S-phase arrest along with a significant decrease in the number of cells in G_0_/G_1_ phase was observed simultaneously in MDA-MB-231 cells treated with either drug alone (Fig. 3). A significant enhancement of S-phase arrest was further observed in the combined treatment, indicating that the cytotoxic effects of As^III^ in combination with Tetra appeared to be due to their ability to induce S-phase arrest. Our findings are also supported by previous studies showing that arsenic induced cell cycle arrest in the S phase in various types of cancer cell lines such as human malignant melanoma cell line and breast cancer cell line [42, 43]. FOXO3a has been implicated in cell cycle arrest leading to growth inhibition via upregulation of p21, p27 and downregulation of Cyclin D1 in various cancers [21–24]. Of note, upregulation of p21 and p27, and downregulation of Cyclin D1 has been closely associated with S-phase arrest in various types of cancer cells including another human TNBC cell line Hs578T [27–30]. In agreement with these previous reports, a clear upregulation of the expression level of FOXO3a and p27 along with decreased Cyclin D1 expression was observed in MDA-MB-231 cells treated with As^III^ combined with Tetra (Fig. 3). We also demonstrated a slight increase in the expression levels of FOXO3a in both single-drug treated and co-administered mice (Fig. 7). More importantly, the modest increase in the expression of p-FOXO3a induced by As^III^ was successfully corrected by the addition of Tetra (Fig. 7), suggesting that the two drugs worked coordinately to downregulate phosphorylated FOXO3a in tumor tissue, consequently maintained its function as tumor suppressor. Similar to the in vitro study, our in vivo study demonstrated that a substantial upregulated p21 expression and downregulated Cyclin D1 expression was also observed in the co-administered mice (Fig. 7). Collectively, our results suggest that antitumor activity of the combined treatment is partially attributed to S-phase arrest associated with the upregulation of FOXO3a, p21, p27 along with the downregulation of p-FOXO3a and Cyclin D1 in MDA-MB-231 cells in vitro and in vivo.

We further demonstrated enhanced LDH release in MDA-MB-231 cells treated with As^III^ in combination with Tetra (Fig. 4), suggesting the involvement of necrosis in the mechanisms of action for the combined treatment. Similarly, a previous report demonstrated that As^III^ induced necrosis through a regulated, Bcl-xL-sensitive mitochondrial pathway in an acute promyelocytic leukemia NB4 cell line [44]. Induction of autophagic cell death by various chemotherapeutic agents has also considered as a potential therapeutic strategy for cancer [17, 36, 37]. In the current study, the cytotoxicity of combining As^III^ with Tetra was partially but significantly abrogated by the addition of wortmannin, a potent autophagy inhibitor [45, 46] (Fig. 5). The activation of autophagy signaling pathway was further confirmed in both vitro and in vivo study, as evidenced by the striking increase in the expression levels of LC3, an autophagic marker [17, 36, 37], along with the activation of the autophagic pathway involving a number of important molecules including phospho-AMPK, phospho-mTOR and Beclin-1 (Figs. 6 and 7). In line with these current findings, Liu et al. [36] and Wang et al. [47] have demonstrated that Tetra functions as a potent agonist for cell autophagy in numerous cancer cells including breast cancer cells. In addition, autophagy has also been demonstrated to partially contribute As^III^-triggered cytocidal effects in human glioma and fibrosarcoma cells in vitro and in vivo [33, 48]. Collectively, our results suggest that besides cell cycle arrest and necrosis induction, autophagic cell death also contributes to the antitumor activity of the combined regimen of As^III^ plus Tetra. Although correlation between autophagy and necrosis has been suggested in various cancer cells [49], results from the current study suggest that the signaling pathway of autophagy and necrosis is independently activated to contribute to the cytotoxicity of the combined treatment based on the fact that the addition of wortmannin showed no influence on the LDH release induced by the combing treatment (Fig. 4).

Recently, an interesting phenomenon referred to as oncology drug repositioning, in which certain drugs conventionally used to treat non-malignant diseases exhibit anticancer effects, has been reported [50]. Study of drug repositioning further suggests that conventional drugs can have antitumor therapeutic effects by activating/suppressing autophagy [50]. Tetra is extensively referenced in the Chinese Pharmacopoeia for its use in the Chinese medicinal system as an analgesic and diuretic agent and also in the treatment of hypertension [51]. Collectively, considering high cost associated with anticancer drug development, As^III^ as well as Tetra, which have long been successfully used in clinic, can probably serve as promising candidates for the development of the novel therapeutic strategies of drug repositioning targeting autophagy to induce breast cancer cell death.

## Conclusions

Our results suggest that Tetra can be a useful combination anticancer agent to enhance therapeutic effect of As^III^, and that development of the combination regimen of As^III^ plus Tetra may offer many benefits to patients with different types of breast cancers. Besides a contribution of S-phase arrest, autophagic cell death and necrosis seemed to independently contribute to the cytocidal effects of the combined regimen of As^III^ and Tetra. Recently, we demonstrated that enhanced intracellular arsenic accumulation (As[i]) along with synergistic cytotoxicity was observed in MCF-7 cells treated with As^III^ combined with Tetra or Ko134, an inhibitor of breast cancer resistance protein (BCRP), suggesting that Tetra or the BCRP inhibitor probably intervened in the occurrence of resistance to arsenic therapy by enhancing the As[i] via modulation of multidrug efflux transporters [12]. In addition, CYP3A4, a major P450 in humans, is involved in the metabolism of half of all currently used drugs including As^III^ [52] and docetaxel, one of the most commonly used chemotherapeutics for breast cancer [53]. Therefore, further studies about the effect of Tetra on As^III^ pharmacokinetics need to be launched in order to provide direct evidence for their clinical use.

## Abbreviations

As^III^: trivalent arsenic (arsenite)
Tetra: tetrandrine
p21: p21 Waf1/Cip1
p27: p27 Kip1
FOXO3a: forkhead box transcription factor 3a
phospho-FOXO3a: phosphorylated FOXO3a
TNBC: triple negative breast cancer
ER: estrogen receptor
HER2: epidermal growth factor receptor-2
LDH: lactate dehydrogenase
As[i]: intracellular arsenic accumulation
BCRP: ABCG2/breast cancer resistance protein
phospho-AMPK: phosphorylated AMP-activated protein kinase
phospho-mTOR: phosphorylated mammalian target of rapamycin
